# The patterns of lifestyle, metabolic status, and obesity among hypertensive Korean patients: a latent class analysis

**DOI:** 10.4178/epih.e2020061

**Published:** 2020-08-31

**Authors:** Suyoung Kim, Seon Cho, Eun-Hee Nah

**Affiliations:** Health Promotion Research Institute, Korea Association of Health Promotion, Seoul, Korea

**Keywords:** Hypertension, Life style, Obesity, Metabolic diseases, Latent class analysis

## Abstract

**OBJECTIVES:**

This study aimed to identify latent classes in hypertensive patients based on the clustering of factors including lifestyle risk factors, metabolic risk factors, and obesity in each sex.

**METHODS:**

This cross-sectional study included 102,780 male and 103,710 female hypertensive patients who underwent health check-ups at 16 centers in Korea, in 2018. A latent class analysis approach was used to identify subgroups of hypertensive patients. Multinomial logistic regression was performed to examine the association between latent classes and comorbidities of hypertension.

**RESULTS:**

A four-class model provided the best fit for each sex. The following latent classes were identified: Class I (male: 16.9%, female: 1.7%; high risk of lifestyle behaviors [HB] with metabolic disorders and obesity [MO]), Class II (male: 32.4%, female: 47.1%; low risk of lifestyle behaviors [LB] with MO), Class III (male: 15.3%, female: 1.8%; HB with metabolic disorders and normal weight [MNW]), Class IV (male: 35.5%, female: 49.4%; LB with MNW). Lifestyle patterns in the latent classes were classified as high-risk or low-risk according to smoking and high-risk drinking among male, and presented complex patterns including physical inactivity alone or in combination with other factors, among female. Stage 2 hypertensive or diabetic individuals were likely to belong to classes including obesity (HB-MO, LB-MO) in both sexes, and additionally belonged to the HB-MNW class in male.

**CONCLUSIONS:**

Metabolic disorders were included in all latent classes, with or without lifestyle risk factors and obesity. Hypertensive females need to manage obesity, and hypertensive males need to manage lifestyle risk factors and obesity. Sex-specific lifestyle behaviors are important for controlling hypertension.

## INTRODUCTION

According to the World Health Organization (WHO), the prevalence of hypertension (HTN) worldwide is 25% in male and 20% in female; less than 20% of these individuals manage their HTN [1]. High blood pressure (BP) is a major risk factor for cardio-cerebrovascular disease and chronic kidney disease, is commonly accompanied by diabetes, liver disease, and cancer (which complicates disease treatment [2,3]), and is a major cause of premature death. Thus, management of high BP is important to prevent disease and lower disease mortality.

Management of BP by lifestyle improvement is recommended and as a supplementary treatment method for the early treatment of hypertensive patients [4]. In intervention studies on stage 1 hypertensive patients, reduction in mean BP and management of HTN were confirmed after lifestyle improvements, in line with other similar studies [4-6]. Modified health behaviors in BP management include heavy drinking, smoking, inadequate physical activity (PA), and excess salt consumption. Other demographic factors such as sex, age, and race, and metabolic status including obesity are also important in managing high BP [5-8]. Metabolic abnormalities and obesity are important components of the metabolic syndrome along with HTN, and mutual influences should be considered in determining HTN risk.

Lifestyle risk factors appear clustered in individuals, and the combination of risk factors may have synergistic effects on disease occurrence. Therefore, an approach based on multiple behavioral characteristics is important in disease management [6,9]. Previous studies on management of HTN have considered the clustered pattern of lifestyle behaviors, and all risk behaviors were numbered/scored or defined as all possible combinations [6,9,10]. However, this complicates interpretation of the results, and has limitations in identifying specific lifestyle factors/patterns. For effective HTN management, it is necessary to seek measures that reflect the homogeneous characteristics as well as the individual influences of the related factors. Latent class analysis (LCA) provides useful information to analyze the clustered characteristics of health risk behaviors. This is used to cluster the subjects to identify homogeneous and mutually exclusive subgroups that exist within subject groups. This method reduces and summarizes multiple categorial data.

LCA provides more information about health status and lifestyle behaviors, which help to identify preventive effect to improve prognosis in subjects with disease [11]. LCA was applied for the purpose of high BP management in studies by Ghanbari et al. [12] who studied obesity and lifestyle patterns of hypertensive patients, and by Trivedi et al. [13] who have assessed adherence patterns in patients, focusing on the hypertensive guidelines. In these studies, latent classes were classified based on lifestyle behaviors and obesity, but not metabolic status. Metabolic abnormalities and obesity play an important role in elevating BP. Hypertensive patients with metabolic abnormalities show changes in cardiovascular or renal function in the preclinical stage, which increase the risk of related diseases [14]. Therefore, it is necessary to analyze the patterns considering lifestyle and metabolic state for BP management.

In this study, LCA was conducted to identify latent classes of hypertensive patients’s risk factors based on lifestyle, obesity, and metabolic status. Characteristics such as age, anthropometric measurements, clinical factors, and disease history were presented, and the distributions of comorbid diseases such as stage 2 HTN, diabetes, and renal and liver function abnormalities were compared by identified subgroups.

## MATERIALS AND METHODS

### Research subjects

**Figure 1.**
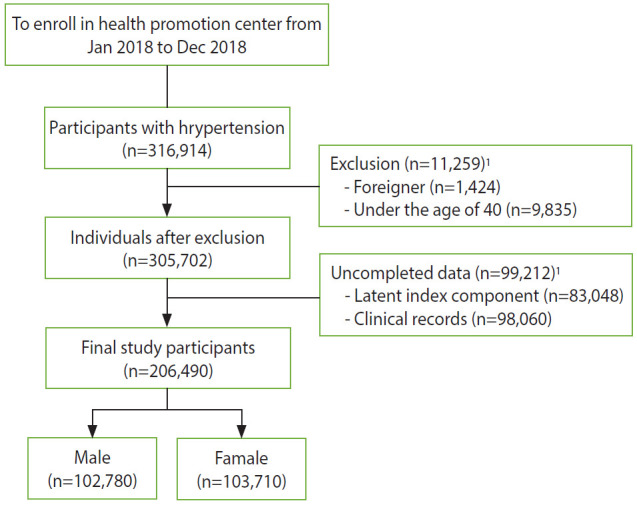
Flow diagram for study participants. ^1^Individuals after duplicate removed.

This cross-sectional study included hypertensive patients aged > 40 years who visited 16 health check-up centers in 13 regions of Korea from January 2018 to December 2018. This study included those who were diagnosed with HTN, were taking anti-hypertensive drugs at the interview, or had a systolic blood pressure (SBP) of > 140 mmHg/a diastolic blood pressure (DBP) of > 90 mmHg [15]. Among those who completed the consent form, non-Korean or individuals who did not complete the questionnaire survey or clinical examination items for the study were excluded. A total of 206,490 subjects were selected (Figure 1).

### Latent class indicators and covariates

Latent class indicators were lifestyle including uncontrolled BP, smoking, high-risk drinking, insufficient PA, and obesity and metabolic abnormalities. Uncontrolled BP was defined as SBP > 140 mmHg/DBP > 90 mmHg. A smoking status was defined as past or current smoker. High-risk drinking was defined as consumption of 7 glasses and 5 glasses of alcohol per time for male and female respectively, and more than twice a week [16]. According to the WHO standard, one minute of vigorous PA was equated to two minutes of moderate PA. Insufficient PA was designated if the total PA time per week was less than 150 minutes [17]. Obesity (body mass index [BMI] ≥ 25 kg/m^2^) and abdominal obesity (waist circumference ≥ 90 cm in male and ≥ 85 cm in female) were considered for obesity status. One or more of high triglycerides (≥ 150 mg/dL), low high-density lipoprotein (HDL) cholesterol (male: < 40 mg/dL; female: < 50 mg/dL), and high fasting blood glucose (≥ 100 mg/dL) were used to designate metabolic abnormality using National Cholesterol Education Program Adult Treatment Panel III.

Age, history of cardiovascular disease, anti-hypertensive drug use, and family history of HTN were considered as covariates for the identified classes based on answers to a health questionnaire, Stage 2 HTN (SBP≥ 160 mmHg/DBP ≥ 100 mmHg), diabetes, and renal and liver dysfunction were included as comorbidities in the latent class. Diabetes mellitus was defined as diagnosis of diabetes, taking antidiabetic drugs or a fasting blood glucose level ≥ 126 mg/dL. Renal dysfunction was defined serum creatinine > 1.5 mg/dL, estimated glomerular filtration rate (e-GFR) < 60 mL/min/1.73 m^2^, or urine protein ≥ 1+. Liver dysfunction was defined aspartate transaminase (AST) ≥ 51 IU/L, alanine transaminase (ALT) ≥ 46 IU/L or gamma-glutamyl transpeptidase (γ-GTP) ≥ 78 IU/L in male/≥ 46 IU/L in female.

### Anthropometric measurements and clinical examinations

BP was measured with an automatic sphygmomanometer. In the case of SBP ≥ 140 mmHg/DBP ≥ 90 mmHg, re-measurements were performed using an aneroid manual sphygmomanometer in accordance with the national health examination standards. BMI was calculated as weight (kg)/height (m)^2^ using an automatic body measuring instrument.

Blood chemistry tests for fasting blood glucose, triglycerides, HDL cholesterol, low-density lipoprotein (LDL) cholesterol. AST, ALT, γ-GTP, and serum creatinine were measured by an enzyme method using the Hitachi 7600 (Hitachi, Nakai, Japan). GFR was calculated by the Modification of Diet in Renal Disease equation, and the urine protein level was measured by the dip-and-read method.

### Statistical analysis

The number of latent classes was determined by LCA, using 7 latent class indicators, and each class was classified based on the probability of each indicator. To determine the optimal number of latent classes, model fit of each latent class was sequentially compared while progressively increasing the number of classes. Akaike information criterion (AIC), Bayesian information criterion (BIC), consistent AIC, and adjusted BIC were comprehensively evaluated for model fit, and a lower value represented a better fit. In addition, entropy was presented to assess the precision of classifying latent class membership in each model; an entropy value closer to 1 indicated a greater precision of classification. The optimal number of latent classes was selected by considering the goodness-of-fit and entropy, the probability (> 1%) that each class contains an entity, interpretability and parsimony [18].

Analysis of variance, t-test, and chi-square tests were performed according to the data type to compare characteristics by sex or derived latent classes. The Scheffe’s method was used for post-hoc testing. A multinomial logistic regression analysis was performed with the low-risk class as the reference in the derived latent class, and the relationship with the state of comorbid diseases was estimated. The SAS version 9.4 (SAS Institute Inc., Cary, NC, USA) program was used for analysis, and the significance level was set to 0.05.

### Ethics statement

This study was conducted with the approval of the Institutional Review Board (IRB) of the Korea Association of Health Promotion (IRB No. 130750-201907-HR-020). Informed consent was confirmed by the IRB.

## RESULTS

### Clinical and disease history characteristics according to sex

Among the study population, 49.8% were male, and 50.2% were female. Anthropometric measurements excluding SBP, clinical tests, and disease history characteristics showed significant sexrelated differences. The mean age was higher in female (61.9±10.5 vs. 64.6±9.2 years), and clinical tests excluding cholesterol level and anthropometric measurements were generally at a higher level in male. A family history of HTN was more frequently observed in female than in male (29.2 vs. 34.2%); history of disease was higher in male than in female (10.3 vs. 7.7%) (Table 1).

All latent class indicators showed significant sex-related differences (Table 1), and their levels were higher in male. However, insufficient PA (42.3 vs. 51.7%) and abdominal obesity (42.1 vs. 43.2%) were higher in female than in male. Smoking, drinking, and insufficient PA showed relatively large sex-related differences (p<0.001 for each). To analyze patterns reflecting sex-related differences in lifestyle, obesity, and metabolic status, latent classes were estimated by dividing sex.

### Classification of latent classes for risk factors in hypertensive patients

To classify the latent classes in hypertensive patients, the number of classes was progressively increased to six, and the model fit indexes AIC, BIC, consistent AIC, and adjusted BIC were compared (Table 2). The model fit index for male decreased as more classes were included in the model, and a slow decline was observed beyond a 4-class model. In the 4-class model, the entropy was 0.83, and the probability of membership in each latent class was > 1%. Thus, this was selected as the final model. An inverse relationship between the number of classes and the model fit index was observed for female as well, and a slow decline was observed beyond a 5-class model. In the 5-class model, the entropy was 0.74, and the membership probability was > 1%. However, three of the 5-class were at a low level of < 11%; thus the number of latent classes was reduced. After a comprehensive consideration of the simplicity of the model, convenience of interpretation, and level of entropy, a 4-class model was selected.

Latent class models classified in male and female were categorized based on the response pattern of the indicator variables in each class. The model divided classes considering high-risk/low-risk lifestyle, metabolic abnormalities, and obesity/normal weight (Table 3). Regarding lifestyle risk level in male, the low-risk subgroups included only smoking and the high-risk subgroups including smoking and high-risk drinking. According to lifestyle risk level, female was classified into a low-risk subgroups that included insufficient PA alone or nothing of all factors, and a high-risk subgroups that included a combination of uncontrolled BP, high-risk drinking, smoking, and insufficient PA. Of the total male participants, 16.9%, 32.4%, 15.3%, and 35.5% were classified into high-risk of lifestyle behaviors with metabolic disorders and obesity (HB-MO), low-risk of lifestyle behaviors with metabolic disorders and obesity (LB-MO), high-risk of lifestyle behaviors with metabolic disorders and normal weight (HB-MNW), and low-risk of lifestyle behaviors with metabolic disorders and normal weight (LB-MNW), respectively. Among female participants, 1.7%, 47.1%, 1.8%, and 49.4% were classified into the HB-MO, LB-MO, HB-MNW, and LB-MNW, respectively.

### Comparison of characteristics among latent classes and related factors

Age, anthropometric measurements, history of HTN or cardiocerebrovascular disease, family history of HTN, and comorbidities showed significant differences among the 4 latent classes in each sex (Table 4). Both male and female in the HB-MO had a lower mean age and a higher mean BP. The HB-MO also had a high probability of family history of HTN, and the high prevalence of stage 2 HTN and liver dysfunction (p<0.001 for each). In the LB-MO, the prevalences of diabetes, renal dysfunction, and a history of cardio-cerebrovascular disease were high. In male, history of disease was highly prevalent in the LB-MNW. Anti-hypertensive drug intake was high in the LB-MO in both male and female. By comparison, a large proportion of male in the HB-MNW and female in the HB-MO class were unaware of having HTN.

Multinomial logistic regression analysis was performed using the LB-MNW as the reference, and factors related to the latent class of HTN were evaluated (Table 5). Those who were taking anti-hypertensive drugs, and those with stage 2 HTN were more likely to be included in the obese classese (HB-MO, LB-MO); In male, these were significantly more likely to be included in the high-risk lifestyle (HB-MNW) and the obese classes. Diabetes had similar characteristics to stage 2 HTN as described above, and both these comorbidities were most likely to be included in the HB-MO. In female, individuals with diabetes were most likely to be included in the LB-MO (odd ratio [OR], 1.57; 95% confidence interval [CI], 1.52 to 1.61). Renal dysfunction and history of disease were more likely to occur in the LB-MO, whereas renal dysfunction was less likely to occur in the HB-MO and HB-MNW when history of disease was less likely to occur or insignificant (OR [95% CI] of LB-MO: renal dysfunction - male: 1.08 [1.03 to 1.13], female: 1.12 [1.07 to 1.17]; history of disease - male: 1.26 [1.21 to 1.30], female: 1.14 [1.10 to 1.18]). Individuals with liver dysfunction were most likely to be included in the HB-MO, and HB-NMW and LB-MO in sequence.

## DISCUSSION

To identify mutually exclusive patterns in hypertensive patients, latent classes based on lifestyle, metabolic abnormalities, and obesity were analyzed, and four latent classes were distinguished for each sex. Metabolic abnormalities were included in all classes, and four classes were designated as follows: HB-MO, LB-MO, HB-MNW, and the LB-MNW. In male, smoking was included in all classes, and high-risk drinking played a role as an indicator to distinguish risk lifestyle behaviors. In contrast, insufficient PA was included in all classes alone or in combination with other lifestyle factors for female, except in the LB-MNW.

Previous studies on latent class delineation in hypertensive patients have classified the cluster pattern of risk factors for HTN into 2-3 classes by risk level [12,13]. Those with uncontrolled BP level were categorized as the high-risk class; insufficient PA was also included as an important classification factor, similar to that for the female in this study. However, previous studies did not include sex-related differences, and only considered lifestyle behaviors as latent class indicators. Sex-related differences in health risk behavior patterns play an important role in the incidence and progression of chronic diseases including HTN [19]. This study identified the different lifestyle characteristics in latent classes by sex, and explained the differences in clinical health characteristics according to heterogeneous subgroups combined with metabolic abnormalities and obesity.

A previous study of lifestyle patterns in the general population reported that patterns in male primarily included drinking and smoking. Also, females were classified based on insufficient PA alone or in combination with other factors [20]. In this study, 2 out of 3 males were distributed into the low-risk lifestyle subgroups including only high-risk drinking, and 96.5% of females were distributed into the low-risk lifestyle subgroups including only insufficient PA or nothing of all factors. Hypertensive patients were mainly identified to distribute in the low-risk lifestyle behavior subgroups compared to the general population. In particular, half of females were distributed in classes without health risk behavior factors. This suggests that there is a more pronounced tendency in hypertensive female to improve their lifestyle. This finding is consistent with the reports of previous studies where female showed higher levels of lifestyle improvement to manage high BP [21,22].

Those who took anti-hypertensive drugs were more likely to be included in an obese class (HB-MO or LB-MO) regardless of their lifestyle risk level. In previous studies, the use of anti-hypertensive drugs was not related to maintaining a normal weight, but was related to obesity [23,24]. The relationship between anti-hypertensive drug use and obesity may be explained by the dependence on drugs in hypertensive patients. Kim & Kong [22] suggested that anti-hypertensive drug use may cause patients to neglect weight management. In contrast, those who did not take anti-hypertensive drugs and were aware of their high BP status, showed a tendency to improve their lifestyle for managing BP [23]. Studies from Europe, Korea, and Spain have reported a difference in drinking and smoking status depending on awareness of HTN [22,25]. These findings suggest that disease awareness is important for transitioning to a healthy lifestyle and maintaining a normal weight. In this study, 16.5% of individuals did not have a HTN diagnosis and were unaware of HTN, suggesting that awareness of HTN is needed for effective BP management.

Independent effect of HTN has been studied by defining metabolically unhealthy normal weight (MUNW) and metabolically healthy obesity (MHO) phenotypes which take into account the major risk factors for HTN [26-29]. Kang et al. [26] and Ryoo et al. [27] (prospective studies in Korea), as well as Tian et al. [28] (cross-sectional study from China) reported that the HTN risk in the MHO and MUNW was significantly higher compared to that of the metabolically healthy normal weight. Moreover, obesity and metabolic status played an independent role in the increased risk of HTN. In contrast, the purpose of this study was to identify the clustered characteristics among hypertensive patients, and metabolic abnormalities were included in all classes. Thus, metabolically healthy obesity classes were not distinguished. In a cohort study of incidence of HTN by Lee et al. [29], many individuals in the MHO moved to metabolically unhealthy during the 8-year follow-up period, and 56.5% of the MHO who developed HTN were metabolically unhealthy at diagnosis. These reports are in line with this study, in which metabolic abnormalities rather than obesity are emphasized in hypertensive patients. Obesity and metabolic abnormalities can be considered as independent predictors of HTN; however, these findings reflect that improving metabolic abnormalities is more important to manage HTN.

lifestyle risk behavior and obesity was independently associated with high BP. Obesity in female was associated with stage 2 HTN in prior to lifestyle risk behaviors. In comparison, it was significantly related to high BP even in the high-risk lifestyle subgroups with normal weight as well as obesity in male. Previous studies have confirmed that HTN and obesity are more closely related in female [30-32]. Faulkner & Belin de Chantemèle [32] reported that the risk of HTN in female increased after menopause, and even before menopause, BP was closely related to BMI in obese female. Unhealthy behavior in male showed high influence than obesity on HTN. In this study, smoking was included in all latent classes of male, and high-risk lifestyles were defined according to drinking status. Thus, high-risk and low-risk lifestyles differed mainly in the drinking status. In a meta-analysis by Roerecke et al. [33], the relationship between alcohol consumption and HTN was more significant in male than in female. Choi et al. [34] analyzed the National Health and Nutrition Examination Survey data and reported that drinking significantly interfered with BP management in male. Sex-related differences was an important factor in managing the risk factors of HTN, and a differential approach was required with focus on obesity management in female, and lifestyle improvement and obesity management in male. The LB-MO was significantly more likely to include a history of cardiocerebrovascular disease, awareness of HTN and comorbidities of HTN. Due to the cross-sectional design of the study, it was unclear whether the lifestyle status at the time of investigation was temporary or permanent. However, obesity was related to the incidence of comorbidities even for low-risk lifestyle status; this may complicate the treatment process.

There are several limitations in this study. First, due to the cross-sectional study design, it was not possible to identify a longitudinal relationship between HTN awareness, anti-hypertensive drug use, and lifestyle modification, and a causal relationship between lifestyle characteristics and comorbidity. Second, HTN was defined based on current BP status or survey response results rather than medical records. However, our analysis included individuals who were hypertensive by inclusion criteria, but were undiagnosed and were unaware of their HTN status. Third, Although the same sphygmomanometer models were not used in 16 examination centers, attempts were made to reduce errors in BP measurements through the standardized guideline and periodic education. Fourth, there are limits that routine national health check-up data collected and used retrospectively. The regional characteristics of the 16 check-up centers may be heterogeneous, and volunteer bias may have existed, as voluntary participants were included in the study. However, the representation of this data was proved in comparison to the national health examination data, in Noh et al. [35]. Thus, it can be seen as representing the general public. Also, as data were collected retrospectively, HTN-related factors such as duration of HTN, salt intake and period of smoking cessation were not included in the analysis [7,21,36]. Therefore, if these factors can be reflected in latent classes, detailed approaches such as lifestyle modification and comorbidity management strategies would be possible.

This study is meaningful as it has identified clustered characteristics including lifestyle factors, metabolic status, and obesity which are closely related to BP management, using large-scale data from hypertensive patients. This study also identified factors that distinguish the defined latent classes. Metabolic abnormalities were included in all latent classes regardless of lifestyle and obesity. Thus, an approach that considers metabolic status should be emphasized in order to effectively manage hypertensive patients. In addition to the general guidelines for managing HTN, female should manage obesity, while male should manage both obesity and their lifestyles. Furthermore, different HTN management strategies that reflect the heterogeneous characteristics of lifestyle patterns according to sex are needed.

## Figures and Tables

**Table 1.** Characteristics of study subjects according to sex

Characteristics	Male (n=102,780)	Female (n=103,710)	p-value
Age (yr)	61.9±10.5	64.6±9.2	<0.001
Anthropometric measurement			
SBP (mmHg)	132.7±15.1	132.9±15.7	0.077
DBP (mmHg)	82.3±10.7	79.9±10.0	<0.001
Waist (cm)	88.7±13.9	83.8±11.2	<0.001
BMI (kg/m²)	25.6±3.1	25.5±3.5	<0.001
Clinical factors			
TG (mg/dL)	155.8±117.9	127±77.5	<0.001
HDL cholesterol (mg/dL)	49.6±12.0	55.3±12.8	<0.001
LDL cholesterol (mg/dL)	105.6±35.8	111.8±36.6	<0.001
FBS (mg/dL)	112.9±30.0	107.1±26.1	<0.001
AST (IU/L)	30.9±21.7	27.7±16.6	<0.001
ALT (IU/L)	30.4±25.4	24.0±19.3	<0.001
γ-GTP (IU/L)	60.4±88.9	30.8±40.3	<0.001
Serum creatinine (mg/dL)	1.12±0.38	0.86±0.27	<0.001
e-GFR (mL/min/1.73 m²)	74.9±15.8	74.5±15.8	<0.001
Urine protein (+)	7,399 (7.2)	3,938 (3.8)	<0.001
History of HTN			
No awareness	18,947 (18.4)	15,075 (14.5)	<0.001
Diagnosis	4,257 (4.1)	2,581 (2.5)	
Diagnosis+medication	79,574 (77.4)	86,054 (83.0)	
History of disease			
Overall	10,567 (10.3)	8,026 (7.7)	<0.001
Stroke	3,746 (3.6)	2,870 (2.8)	<0.001
Heart disease	7,426 (7.2)	5,570 (5.4)	<0.001
Family history of HTN	29,958 (29.2)	35,483 (34.2)	<0.001
Latent class indicators			
Lifestyle behavior			
Uncontrolled blood pressure	45,286 (44.1)	41,918 (40.4)	<0.001
Smoking: past or current	74,786 (72.8)	3,797 (3.7)	<0.001
High-risk alcohol drinking	33,129 (32.2)	3,546 (3.4)	<0.001
Insufficient physical activity	43,495 (42.3)	53,636 (51.7)	<0.001
Metabolic status			
Metabolic risk (≥1 component)	85,222 (82.9)	84,121 (81.1)	<0.001
Obesity			
Obesity	57,594 (56.0)	53,875 (52.0)	<0.001
Abdominal obesity	43,231 (42.1)	44,831 (43.2)	<0.001

Values are presented as mean±standard deviation or number (%). SBP, systolic blood pressure; DBP, diastolic blood pressure; TG, triglycerides; HDL, high-density lipoprotein; LDL, low-density lipoprotein; FBS, fasting blood glucose; AST, aspartate transaminase; ALT, alanine transaminase; γ-GTP, gamma-glutamic transpeptidase; e-GFR, estimated glomerular filtration rate; HTN, hypertension.

**Table 2.** Comparison of model fit statistics for the latent class models by sex

Sex	Classes	Log-likelihood	G²	AIC	BIC	CAIC	Adjusted BIC	Entropy	df
Male	2	-431,603.15	4,081.38	4,111.38	4,254.48	4,269.48	4,206.81	0.72	112
	3	-430,447.66	1,770.39	1,816.39	2,035.82	2,058.82	1,962.73	0.82	104
	4	-429,942.79	760.65	822.65	1,118.40	1,149.40	1,019.88	0.83	96
	5	-426,848.17	534.55	612.55	984.62	1,023.62	860.68	0.71	88
	6	-429,671.73	218.53	312.53	760.93	807.93	611.56	0.65	80
Female	2	-344,564.29	2,844.28	2,874.28	3,017.52	3,032.52	2,969.85	0.75	112
	3	-343,756.32	1,228.34	1,274.34	1,493.98	1,516.98	1,420.89	0.84	104
	4	-343,462.67	641.03	703.03	999.06	1,030.06	900.54	0.87	96
	5	-343,285.91	287.51	365.51	737.94	776.94	613.99	0.74	88
	6	-343,227.77	171.24	265.24	714.06	761.06	564.69	0.72	80

AIC, Akaike information criterion; BIC, Bayesian information criterion; CAIC, consistent Akaike information criterion; df, degree of freedom.

**Table 3.** Prevalence of lifestyle behaviors, metabolic risk, and obesity indicators, and conditional probabilities of class membership in the class mode

Obesity status	Latent class			
	Class I (HB-MO)	Class II (LB-MO)	Class III (HB-MNW)	Class IV (LB-MNW)
Male				
Latent class prevalence (%)	16.9	32.4	15.3	35.5
Lifestyle behaviors				
Uncontrolled blood pressure	0.484	0.437	0.469	0.411
Past or current smoking	0.829	0.695	0.842	0.660
High-risk alcohol drinking	0.983	0.011	0.994	0.002
Insufficient physical activity	0.441	0.454	0.405	0.394
Metabolic status				
Metabolic risk (≥1 component)	0.902	0.878	0.801	0.762
Obesity status				
Obesity (BMI≥25 kg/m²)	0.937	0.918	0.229	0.197
Abdominal obesity (male: WC≥90 cm; female; WC≥85 cm)	0.843	0.821	0.026	0.024
Female				
Latent class prevalence (%)	1.7	47.1	1.8	49.4
Lifestyle behaviors				
Uncontrolled blood pressure	0.510	0.418	0.326	0.390
Past or current smoking	0.209	0.034	0.961	0.000
High-risk alcohol drinking	0.974	0.000	0.255	0.026
Insufficient physical activity	0.558	0.560	0.550	0.474
Metabolic status				
Metabolic risk (≥1 component)	0.797	0.878	0.751	0.750
Obesity status				
Obesity (BMI≥25 kg/m²)	0.891	0.888	0.067	0.171
Abdominal obesity (male: WC≥90 cm; female: WC≥85 cm)	0.872	0.856	0.041	0.027

HP-MO, high-risk lifestyle behavior with metabolic disorder and obesity; LP-MO, low-risk lifestyle behavior with metabolic disorder and obesity; HP-MNW, high-risk lifestyle behavior with metabolic disorder and normal weight; LP-MNW, low-risk lifestyle behavior with metabolic disorder and normal weight; BMI, body mass index; WC, waist circumference.

**Table 4.** Differences in characteristics of anthropometric measurements, clinical factors, and disease history among the four latent classes

Characteristics	Class I (HB-MO)	Class II (LB-MO)	Class III (HB-MNW)	Class IV (LB-MNW)	p-value	Multiple comparison
Male						
Age (yr)	57.3±9.6	62.5±10.8	58.6±9.1	64.7±10.1	<0.001	I<II<III<IV
Anthropometric measurement						
SBP (mmHg)	133.5±15.0	132.9±14.9	132.9±15.4	132.3±15.3	<0.001	IV<II, III<I
DBP (mmHg)	84.5±10.7	82.2±10.5	83.7±10.8	81.0±10.5	<0.001	IV<II<III<I
History of HTN						-
No awareness	2,867 (19.0)	4,625 (15)	4,457 (24.8)	6,998 (18.0)	<0.001	
Diagnosis	695 (4.6)	983 (3.2)	953 (5.3)	1,626 (4.2)		
Diagnosis+medication	11,562 (76.5)	25,150 (81.8)	12,595 (70.0)	30,267 (77.8)		
History of disease						-
Overall	934 (6.2)	3906 (12.7)	1003 (5.6)	4724 (12.2)	<0.001	
Stroke	282 (1.9)	1347 (4.4)	322 (1.8)	1795 (4.6)	<0.001	
Heart disease	698 (4.6)	2798 (9.1)	705 (3.9)	3225 (8.3)	<0.001	
Family history of HTN	6,445 (42.6)	11,665 (37.9)	7,039 (39.1)	13,769 (35.4)	<0.001	-
Comorbidities						-
Stage 2 HTN	1,440 (9.5)	2,311 (7.5)	1,704 (9.5)	2,681 (6.9)	<0.001	
Diabetes	5,458 (36.1)	11,758 (38.2)	5,370 (29.8)	12,133 (31.2)	<0.001	
Renal dysfunction	2,333 (15.4)	6,955 (22.6)	2,253 (12.5)	7,525 (19.4)	<0.001	
Liver dysfunction	7,273 (48.1)	8,161 (26.5)	6,414 (35.6)	5,411 (13.9)	<0.001	
Female						
Age (yr)	56.0±8.7	65.5±9.2	59.5±9.8	64.2±9.1	<0.001	I<III<IV<II
Anthropometric measurement						
SBP (mmHg)	135.2±16.1	134.6±15.5	127.7±16.4	131.5±15.6	<0.001	III<IV<II<I
DBP (mmHg)	84.7±10.8	80.5±9.8	79.1±11	79.4±10.0	<0.001	III,IV<II<I
History of HTN						-
No awareness	407 (23.6)	5891 (12.9)	263 (16.5)	8,514 (15.6)	<0.001	
Diagnosis	49 (2.8)	864 (1.9)	76 (4.8)	1,592 (2.9)		
Diagnosis+medication	1,272 (73.6)	39,044 (85.3)	1,258 (78.8)	44,480 (81.5)		
History of disease						-
Overall	64 (3.7)	4,027 (8.8)	115 (7.2)	3,820 (7.0)	<0.001	
Stroke	22 (1.3)	1,403 (3.1)	50 (3.1)	1,395 (2.6)	<0.001	
Heart disease	46 (2.7)	2,829 (6.2)	73 (4.6)	2,622 (4.8)	<0.001	
Family history of HTN	856 (49.5)	18,509 (40.4)	799 (50.0)	24,001 (44.0)	<0.001	-
Comorbidities						-
Stage 2 HTN	199 (11.5)	3,164 (6.9)	89 (5.6)	3,065 (5.6)	<0.001	
Diabetes	401 (23.2)	15,427 (33.7)	349 (21.9)	12,364 (22.7)	<0.001	
Renal dysfunction	148 (8.6)	9,773 (21.3)	227 (14.2)	9,296 (17.0)	<0.001	
Liver dysfunction	714 (41.3)	9,202 (20.1)	406 (25.4)	6,309 (11.6)	<0.001	

Values are presented as mean±standard deviation or number (%). HP-MO, high-risk lifestyle behavior with metabolic disorder and obesity; LP-MO, low-risk lifestyle behavior with metabolic disorder and obesity; HP-MNW, high-risk lifestyle behavior with metabolic disorder and normal weight; LP-MNW, low-risk lifestyle behavior with metabolic disorder and normal weight; SBP, systolic blood pressure; DBP, diastolic blood pressure; HTN, hypertension.

**Table 5.** Associations between latent class membership and demographic or clinical characteristics

Characteristics	Latent class			
	Class I (HB-MO)	Class II (LB-MO)	Class III (HB-MNW)	Class IV (LB-MNW)
Male				
Age (per 10 yr increase)	0.53 (0.52, 0.54)^*^	0.79 (0.78, 0.81)^*^	0.62 (0.61, 0.63)^*^	1.00 (reference)
History of disease	0.64 (0.59, 0.69)^*^	1.08 (1.03, 1.13)^*^	0.58 (0.54, 0.62)^*^	1.00 (reference)
Medication for HTN	1.50 (1.43, 1.58)^*^	1.43 (1.37, 1.49)^*^	0.99 (0.95, 1.04)	1.00 (reference)
Family history of HTN	1.02 (0.98, 1.06)	0.99 (0.96, 1.02)	0.97 (0.93, 1.00)	1.00 (reference)
Comorbidities				
Stage 2 HTN	1.21 (1.12, 1.30)^*^	1.14 (1.08, 1.22)^*^	1.09 (1.02, 1.17)^*^	1.00 (reference)
Diabetes	1.41 (1.35, 1.47)^*^	1.33 (1.29, 1.37)^*^	1.12 (1.07, 1.16)^*^	1.00 (reference)
Renal dysfunction	0.95 (0.90, 1.00)	1.26 (1.21, 1.30)^*^	0.76 (0.72, 0.80)^*^	1.00 (reference)
Liver dysfunction	4.32 (4.13, 4.51)^*^	2.03 (1.96, 2.11)^*^	2.72 (2.61, 2.84)^*^	1.00 (reference)
Female				
Age (per 10 yr increase)	0.40 (0.38, 0.43)^*^	1.12 (1.11, 1.14)^*^	0.58 (0.55, 0.61)^*^	1.00 (reference)
History of disease	0.85 (0.66, 1.10)	1.12 (1.07, 1.17)^*^	1.36 (1.12, 1.65)^*^	1.00 (reference)
Medication for HTN	1.19 (1.05, 1.35)^*^	1.18 (1.14, 1.23)^*^	1.10 (0.96, 1.25)	1.00 (reference)
Family history of HTN	0.92 (0.83, 1.01)	0.91 (0.88, 0.93)^*^	1.04 (0.94, 1.16)	1.00 (reference)
Comorbidities				
Stage 2 HTN	1.74 (1.47, 2.05)^*^	1.34 (1.27, 1.42)^*^	0.89 (0.71, 1.11)	1.00 (reference)
Diabetes	1.15 (1.02, 1.29)^*^	1.57 (1.52, 1.61)^*^	1.00 (0.89, 1.13)	1.00 (reference)
Renal dysfunction	0.70 (0.58, 0.83)^*^	1.14 (1.10, 1.18)^*^	1.06 (0.92, 1.23)	1.00 (reference)
Liver dysfunction	4.42 (4.00, 4.90)^*^	1.90 (1.83, 1.97)^*^	2.32 (2.06, 2.61)^*^	1.00 (reference)

Values are presented as odds ratio (95% confidence interval). HP-MO, high-risk lifestyle behavior with metabolic disorder and obesity; LP-MO, low risk lifestyle behavior with metabolic disorder and obesity; HP-MNW, high-risk lifestyle behavior with metabolic disorder and normal weight; LP-MNW, low-risk lifestyle behavior with metabolic disorder and normal weight; HTN, hypertension.

* p<0.05.

## SUPPLEMENTARY MATERIALS

Korean version is available at http://www.e-epih.org/.
