# Supplementary material for: The patterns of lifestyle, metabolic status, and obesity among hypertensive Korean patients: a latent class analysis
Source: Epidemiol Health. 2020 Aug 31;42:e2020061. doi: 10.4178/epih.e2020061 (PMC7871153; doi:10.4178/epih.e2020061)
Supplement: Supplementary file 1 [file epih-42-e2020061-suppl.docx]

**The patterns of lifestyle, metabolic status and obesity among hypertensive patients: a latent class analysis**

**고혈압 환자의 생활습관과 대사적 특성 및 비만 패턴: 잠재계층분석**

**Suyoung Kim, Seon Cho, Eun-Hee Nah**

**Health Promotion Research Institute, Korea Association of Health Promotion, Seoul, Korea**

**Short title:** The latent classes in hypertension

Correspondence: Eun-Hee Nah

Health Promotion Research Institute, Korea Association of Health

Promotion, 396 Gonghang-daero, Gangseo-gu, Seoul 07649, Korea

E-mail: cellonah@hanmail.net

**Abstract**

Objectives: This study aimed to identify latent classes in hypertensive patients based on the clustering of risk factors including lifestyle risk factors, metabolic risk factors, and obesity in each sex.

Methods: This cross-sectional study included 102,780 male and 103,710 female hypertensive patients who underwent health checkups at 16 centers in Korea, in 2018. A latent class analysis approach was used to identify subgroups of hypertensive patients. Multinomial logistic regression was performed to examine the association between latent classes and comorbidities of hypertension.

Results: A four-class model provided the best fit for each sex. The following latent classes were identified: Class Ⅰ [male:16.9%,female:1.7%; high risk of lifestyle behaviors (HB) with metabolic disorders and obesity (MO)], Class Ⅱ [male:32.4%, female:47.1%; low risk of lifestyle behaviors (LB) with MO], Class Ⅲ [male:15.3%, female:1.8%; HB with metabolic disorders and normal weight (MNW)], Class Ⅳ [male:35.5%, female:49.4%; LB with MNW]. Lifestyle patterns in the latent classes were classified as high- or low-risk according to smoking and high-risk drinking among male, and presented complex patterns including physical inactivity alone or in combination with other factors, among female. Stage 2 hypertensive or diabetic individuals were likely to belong to classes including obesity (HB-MO, LB-MO) in both sexes, and additionally belonged to the HB-MNW class in male.

Conclusions: Metabolic disorders were included in all latent classes, with or without lifestyle risk factors and obesity. Hypertensive female need to manage obesity, and hypertensive male need to manage lifestyle risk factors and obesity. Sex-specific lifestyle behaviors are important for controlling hypertension.

**Key words:** Hypertension, Life style, Obesity, Metabolic disorders, Latent class analysis

**Introduction**

WHO 보고에 따르면 전세계 고혈압 유병 수준은 남성 4명 중 1명, 여성 5명 중 1명으로 추산되며, 이 중 고혈압을 관리하는 비율은 20% 미만 수준이다[1]. 고혈압은 심뇌혈관 질환, 만성 신장질환의 주요 위험요인으로 알려져 있으며, 당뇨병, 간 질환, 암 등에 흔하게 동반된다[2,3]. 또한 조기 사망의 주요 원인으로 작용하므로, 질병의 예방 뿐 만 아니라 질환 사망률을 낮추는데 높은 혈압을 관리하는 것은 중요하다.

생활습관 개선에 의한 혈압 관리는 최적 혈압을 유지하는 것 뿐 만 아니라, 고혈압 환자에서 초기 치료 및 약물복용의 보조 치료 방법으로 추천된다[4]. 1단계 고혈압 환자 대상의 중재연구에서 생활습관 개선으로 평균 혈압의 감소와 고혈압 관리 효과를 확인한 것을 비롯하여, 많은 연구에서 생활습관 개선을 통한 높은 혈압의 관리 효과를 규명하였다[4–6]. 혈압관리에서 조절가능한 생활습관 요인은 과도한 음주, 흡연, 충분하지 않은 신체활동, 짜게먹는 식습관 등이 있고, 그 외 성, 연령, 인종 등의 인구사회학적 요인과 비만을 포함한 대사 상태 등이 있으며, 이들은 고혈압 관리에 중요한 요인으로 작용한다[5–8]. 특히 대사이상과 비만은 고혈압과 함께 대사증후군의 구성요소로서, 고혈압의 위험도를 규명하는데 상호 영향력을 고려해야 한다.

생활습관 위험요인은 개인에서 군집화되어 나타나고, 그 결합된 형태가 질병 발생에 시너지효과를 보이기 때문에, 질환 관리 측면에서 다중 행동특성에 기인한 통합적인 접근이 중요하다[6,9]. 이전 연구에서는 생활습관 행동의 군집 패턴을 고려한 접근을 강조하는 결과를 보고하였으나, 고혈압 건강위험 행동 조합에 대한 모든 경우를 정의하여 비교하거나, 위험행동의 갯수 혹은 점수화하여 그 위험도를 제시하였다[6,9,10]. 이는 결과를 해석하는데 복잡할 뿐 만 아니라, 고혈압 위험수준과 관련된 구체적인 생활습관 요인과 패턴을 확인하는데 한계가 있다. 고혈압의 효과적인 관리를 위해서는 관련 요인들의 개별적인 영향력 뿐만 아니라 동질적인 특징을 반영한 방안을 모색할 필요가 있다. 이러한 건강위험행동 요인의 군집화된 특성을 확인하는데 잠재계층분석(Latent Class Analysis, LCA)은 유용한 정보를 제공한다. 이는 대상자의 자료를 군집화하여 대상자 내 존재하는 동질적이면서 상호배타적인 하위집단을 확인하는데 활용되며, 다중 범주형 자료를 축소 요약하는 방법이다.

LCA는 건강상태와 생활습관을 서술하는데 더 많은 정보를 제공하여 질병 예후를 개선하기 위한 예방적 효과를 알아내는데 도움이 된다[11]. 혈압 관리를 목적으로 LCA를 적용한 연구로는 고혈압 환자의 비만과 생활습관 패턴을 확인한 Ghanbari J 등[12]의 연구와, 고혈압 가이드라인 항목을 중심으로 환자의 이행 패턴을 확인한 Trivedi RB 등[13]의 연구가 있다. 이들은 대체로 비만을 포함한 생활습관을 중심으로 계층을 분류하여, 대상자의 대사 상태를 반영하지 않았다. 대사 이상 상태는 비만과 함께 혈압을 높이는데 중요한 역할을 하며, 대사 이상이 있는 고혈압 환자는 임상전단계에서 심뇌혈관 혹은 신장 기능의 변화를 초래하여 이들 질환발생의 위험을 가중시킨다[14].

본 연구에서는 생활습관, 비만 및 대사상태를 잠재지표로 정의하여 고혈압 환자의 군집화된 잠재집단을 확인하고자 LCA를 실시한다. 또한 확인된 하위 잠재 집단별로 연령, 신체계측, 임상적 요인 및 질환력 등의 특성을 제시하며, 2단계 고혈압, 당뇨병, 신기능 및 간기능 이상 등의 동반질환 분포를 비교하고자 한다.

**Materials and Methods**

**연구 대상자**

본 단면연구(Cross-sectional study)는 2018년 1월부터 12월까지 한국의 13개 지역, 16개 건강검진센터에 건강검진을 목적으로 방문한 40세 이상의 고혈압 환자를 대상으로 하였다. 연구에 포함된 고혈압 환자는 문진 조사에서 고혈압으로 진단을 받았거나 항고혈압제를 복용한다고 응답한 경우, 혹은 수축기 혈압 140mmHg 이상 혹은 이완기 혈압 90mmHg 이상인 경우로 정의했다[15]. 동의서를 작성한 대상자 중, 외국인이거나 연구에 필요한 문진조사 혹은 임상검사 항목을 완료하지 않은 경우는 제외하였으며, 총 206,490명을 최종 대상자로 선정하였다[Fig 1].

(Figure 1 위치)

**잠재계층 지표 및 공변량**

연구대상자의 잠재계층을 분류하기 위한 주요 지표로, 관리되지 않은 혈압, 흡연, 고위험 음주, 부족한 신체활동이 포함된 생활습관과 비만 및 대사이상 상태를 고려하였으며, 모든 지표는 이분형으로 정의하였다. 관리되지 않은 혈압은 수축기 혈압 140mmHg 이상 혹은 이완기 혈압 90mmHg 이상으로 정의하였다. 흡연은 과거 혹은 현재 흡연하는 경우로 평가하였다. 고위험 음주는 지난 1년간의 평균 음주 빈도와 가장 많이 마셨던 하루의 음주량 등을 중심으로, 1회 평균 음주량이 남성 7잔, 여성 5잔 이상이고 주 2회 이상 음주한 경우로 정의하였다[16]. 또한 평소 1주일간 중강도 이상의 신체활동을 실시한 일수와 하루 평균 활동시간을 조사하여 신체활동 수준을 확인하였다. WHO기준에 따라, 고강도 신체활동의 1분을 중등도 신체활동의 2분과 같게 평가하며, 중등도와 고강도 신체활동 시간을 합하여 1주일간 총 신체활동 시간이 150분 미만이면 신체활동이 충분하지 않다고 정의하였다[17]. 비만상태는 비만과 복부비만 두 요인을 고려하였으며, BMI ≥25 kg/m²은 비만으로, 허리둘레 기준 남성 ≥90 cm, 여성 ≥85 cm는 복부비만으로 정의하였다. 대사 이상은 NCEP ATP Ⅲ 기준을 적용하여, 고 중성지방(≥150 mg/dL), 저 고밀도콜레스테롤(남성 <40 mg/dL, 여성 <50 mg/dL), 고 식전혈당(≥100 mg/dL) 중 하나 이상의 항목이 해당되는 경우로 분류하였다.

잠재계층분석으로 유형화된 계층의 공변량으로, 연령, 심혈관 질환의 과거력, 항고혈압제 복용여부, 고혈압 가족력을 고려하였다. 즉, 질환과거력은 건강문진 조사 응답결과, 뇌졸중 혹은 심근경색을 진단받았거나 치료제를 복용한 경우, 가족력은 부모, 형제, 자매 중에 고혈압을 진단받은 경우로 정의하였다. 또한 잠재 집단의 동반질환으로 2단계 고혈압, 당뇨병, 신기능 이상, 간기능 이상을 포함하였다. 2단계 고혈압은 수축기 혈압 ≥160 mmHg 또는 이완기 혈압 ≥100 mmHg인 경우, 당뇨병은 당뇨병을 진단받아 약물을 복용하거나 식전혈당 ≥ 126mg/dL인 경우, 신기능 이상은 혈청크레아티닌 > 1.5 mg/dL, eGFR < 60mL/min/1.73m², 요단백 ≥ 1+ 중 하나라도 해당하는 경우, 간기능 이상은 AST ≥ 51 IU/L, ALT ≥ 46 IU/L, γ-GTP ≥ 78 IU/L (남성) 혹은 ≥46 IU/L (여성) 중 하나라도 해당하는 경우로 정의하였다.

**신체계측 및 임상검사 측정**

혈압은 자동혈압계로 측정하였으며, 국가 건강검진 실시기준에 따라 수축기 혈압 ≥ 140 mmHg 혹은 이완기 혈압 ≥ 90 mmHg인 경우는 아네로이드식 수동혈압계로 재측정하였다. 자동 신체계측기로 측정한 체중(kg)/신장(m)²으로 체질량지수(body mass index, BMI)를 계산하였다.

공복혈당, 중성지방, HDL 콜레스테롤, LDL 콜레스테롤, AST, ALT, γ-GTP, 혈청 크레아티닌 등의 혈액화학검사는 Hitachi 7600 (Hitachi, Nakai, Japan)을 이용하여 효소법으로 측정하였다. 신사구체여과율은 MDRD (modification of diet in renal disease) 방식으로 계산하였으며, 요단백은 시험지법으로 측정하였다.

**통계분석**

잠재계층 지표 7개 항목을 포함한 LCA 분석을 실시하여 잠재계층 수를 결정하며, 각 계층은 지표별 확률에 기반하여 유형화하였다. 최적의 잠재계층 수를 결정하기 위해, 계층의 수를 순차적으로 증가시키면서 각 잠재계층모형의 적합도를 반복적으로 비교하였다. 모형 적합도는 Akaike information criterion (AIC), Bayesian information criterion (BIC)와 Consistent AIC, adjusted BIC를 종합적으로 평가하며, 값이 작을수록 더 적합하다고 판단한다. 또한 각 모형에서 개별 개체들이 잠재계층으로 정확하게 분류되었는지를 평가할 수 있는 Entropy를 제시하였으며, 1에 가까울수록 분류 정확도가 높다고 판단한다. 최적의 잠재계층 수는 적합도 지수와 Entropy 뿐 만 아니라, 각 계층별로 개체가 포함되는 확률(1% 이상)과 해석 가능성, 모형 간결성 등을 함께 고려하여 선택하였다[18].

성별 혹은 LCA로 도출된 잠재계층별 신체계측과 임상적 특성, 질환 과거력 및 가족력 등의 특성을 비교하기 위해 자료의 형태에 따라 T-test, ANOVA와 Chi-square test를 수행하였으며, 사후검정은 Scheffe 방법을 활용하였다. 도출된 잠재계층에서 위험수준이 낮은 계층을 기저집단으로 한 다항로지스틱 회귀분석을 실시하였으며, 2단계 고혈압, 당뇨병, 신기능 및 간기능 이상 등 동반질환 상태와의 관련성을 추정하였다. SAS 9.4 (SAS Institute, Cary, NC, USA) 프로그램을 사용하였으며, 유의수준 0.05를 기준으로 분석하였다.

**Results**

**성별에 따른 임상 및 질환력 특성**

연구대상자 중 남성과 여성의 비율은 각 49.8%, 50.2%로 비슷하였으며, 수축기혈압을 제외한 신체계측, 임상 항목과 질환력 특성은 모두 성별에 따라 유의한 차이를 보였다. 평균 연령은 여성에서 높았으며(61.9 ± 10.5세 vs 64.6 ± 9.2세), 신체계측 항목과 콜레스테롤을 제외한 임상항목에서 전반적으로 남성이 높았다. 고혈압 가족력은 남성보다 여성이 높은데 비해(29.2% vs 34.2%), 질환과거력은 남성에서 높았다(10.3% vs 7.7%) [Table 1].

잠재계층 지표로 고려한 7개 항목은 모두 성별에 따라 유의한 차이를 보였다[Table 1]. 대부분의 항목은 남성에서 높은 수준인데 비해, 부족한 신체활동(42.3% vs 51.7%)과 복부비만(42.1% vs 43.2%)은 여성에서 더 높았다. 흡연과 음주, 부족한 신체활동은 성별간 차이가 비교적 크게 나타났다(각 p-value<0.001). 성별에 따른 생활습관, 비만 및 대사상태 차이를 반영한 패턴을 확인하고자, 남성과 여성을 구분하여 잠재계층을 추정하였다.

(Table 1 위치)

**고혈압 환자의 위험요인 잠재계층유형**

고혈압 환자의 잠재계층유형을 분류하기 위해, 잠재 계층수를 6개까지 증가시키면서 모형 적합도 지수 AIC, BIC, CAIC, adj. BIC를 비교하였다[Table 2]. 남성의 모형 적합도 지수는 많은 계층수를 포함한 모형일수록 감소하였으며, 4계층 모형 이후 더딘 감소추세를 보였다. 4계층 모형에서 Entropy값은 0.83이었으며, 추정된 모형의 각 잠재집단 분포는 모두 1% 이상으로 적절하여 최종 모형으로 선택하였다. 여성 역시 모형 내 잠재 계층수와 모형 적합도 지수의 반비례적 관계를 확인하였으며, 5계층 모형 이후 감소 속도가 줄었다. 5계층 모형에서 Entropy는 0.74였고, 추정된 모형의 각 잠재집단 분포는 모두 1% 이상이었으나, 이 중 3개 집단이 11% 이하의 낮은 수준이었기 때문에 잠재 계층수를 축소하였다. 모형의 간결성과 해석의 편의성, Entropy 수준 등을 종합적으로 고려하여, 4계층 모형을 최종 선택하였다.

(Table 2 위치)

남성과 여성에서 분류된 잠재계층모형은 각 계층에서 지표변수의 반응 패턴에 기반하여 유형화하였으며, 고위험 혹은 저위험 생활습관과 대사이상이면서 비만 혹은 정상체중의 조합으로 구분하였다[Table 3]. 생활습관 위험수준은 남성에서 흡연만 포함된 저위험 계층과 흡연 및 고위험 음주가 함께 포함된 고위험 계층으로 구분하였으며, 여성에서 부족한 신체활동만 포함되거나 생활습관 위험지표가 포함되지 않은 저위험 계층과 관리되지 않은 혈압, 고위험 음주, 흡연, 부족한 신체활동 등이 복합적으로 포함된 고위험 계층으로 구분하였다. 남성은 고위험 생활습관과 대사이상이면서 비만(High risk of lifestyle behaviors with metabolic disorders and obesity; HB-MO) 16.9%, 저위험 생활습관과 대사이상이면서 비만(Low risk of lifestyle behaviors with metabolic disorders and obesity; LB-MO) 32.4%, 고위험 생활습관과 대사이상이면서 정상체중(High risk of lifestyle behaviors with metabolic disorders and normal weight; HB-MNW) 15.3%, 저위험 생활습관과 대사이상이면서 정상체중(Low risk of lifestyle behaviors with metabolic disorders and normal weight; LB-MNW) 35.5%로 계층화 되었으며, 여성은 HB-MO 1.7%, LB-MO 47.1%, HB-MNW 1.8%, LB-MNW 49.4%로 계층화되었다.

(Table 3 위치)

**잠재계층유형간 특성 비교 및 관련요인**

대상자의 연령, 신체계측, 고혈압 및 심뇌혈관 질환 과거력, 고혈압 가족력, 동반질환 등의 특징은 각 성별에서 분류된 4개의 잠재계층 집단 간에 유의한 차이를 보였다[Table 4]. 남성과 여성 모두HB-MO군에서 평균 연령이 낮고, 혈압 평균수준이 높았다. 또한 고혈압 가족력 수준이 높았고, 2단계 고혈압과 간기능 이상 유병률이 높았다(각 p-value<0.001). LB-MO군은 당뇨병과 신기능 이상 유병률이 높았으며, 심뇌혈관계 질환 과거력 비율이 높은 가운데, 남성에서는 LB-MNW군에서도 질환과거력이 높았다. 항고혈압제 복용률은 남성과 여성 모두 LB-MO군에서 높은데 비해, 고혈압을 인지하지 못하는 비율은 남성은 HB-MNW군에서, 여성은 HB-MO군에서 높은 특징을 보였다.

(Table 4 위치)

LB-MNW군을 기저로 다중로지스틱 회귀분석을 실시하여, 고혈압 잠재집단에 관련된 인자를 확인하였다[Table 5]. 항고혈압제를 복용하는 대상자는 비만 집단(HB-MO, LB-MO)일 가능성이 높았다. 2단계 고혈압이 있는 대상자는 비만 집단(HB-MO, LB-MO)에 포함될 가능성이 높았으며, 특히 남성의 경우 비만계층 뿐 만 아니라 고위험 생활습관 계층(HB-MNW)에 포함될 가능성도 유의하게 높았다. 당뇨병은 앞서 설명한 2단계 고혈압과 유사한 특성이었으며, 당뇨병과 2단계 고혈압 모두 HB-MO군에 포함될 가능성이 가장 높았다. 그러나 여성에서 당뇨병이 있는 대상자는 LB-MO에 포함될 가능성이 가장 높았다[OR (95% CI): 1.57 (1.52, 1.61)]. 신기능 이상은 과거 질환력이 있는 경우와 함께 LB-MO에 속할 가능성이 높았으며, 과거 질환력이 포함될 가능성이 낮거나 유의하지 않는 HB-MO군과 HB-MNW군에는 신기능 이상이 포함될 가능성이 역시 높지 않았다[LB-MO군의 OR (95% CI): 신기능 이상 - 남성 1.08 (1.03, 1.13), 여성 1.12 (1.07, 1.17); 과거 질환력 - 남성 1.26 (1.21, 1.30), 여성1.14 (1.10, 1.18)]. 간기능 이상인 대상자는 HB-MO군에 속할 가능성이 가장 높았으며, 고위험 생활습관군(HB-MNW)이거나 비만군(LB-MO)에서도 유의하게 높았다.

(Table 5 위치)

**Discussion**

고혈압 환자에서 상호 배타적인 집단을 확인하기 위해, 생활습관, 대사이상 및 비만에 기반한 잠재계층을 확인한 결과, 각 성별에서 4개 계층으로 분류되었다. 모든 계층에는 대사이상 상태가 포함되었으며, 고위험 생활습관과 비만(HB-MO), 저위험 생활습관과 비만(LB-MO), 고위험 생활습관과 정상체중(HB-MNW), 저위험 생활습관과 정상체중(LB-MNW)으로 유형화하였다. 남성은 모든 계층에 흡연이 포함되었고, 고위험 음주가 생활습관을 구분하는 지표로 작용하였다. 이에 비해, 여성에서는 LB-MNW을 제외한 모든 계층에 부족한 신체활동이 단독 혹은 다른 생활습관 요인과 결합되어 다양하게 구분되었다.

고혈압 환자에서의 잠재계층 분류에 대한 연구로서, 선행연구에서는 고혈압 위험요인의 군집 패턴을 위험수준별로 2-3개의 계층으로 구분하였다[12,13]. 관리되지 않은 혈압을 가진 대상자가 고위험 계층에 분포하였고, 충분하지 않은 신체활동 역시 계층을 구분하는 중요한 요인으로 포함되어, 본 연구의 여성 대상자와 유사한 패턴이었다. 그러나 선행연구는 성별의 구분없이 전체 대상자를 분석하였으며, 잠재계층지표로 단지 생활습관 요인을 고려한 점에서 본 연구와 차이가 있다. 건강위험행동 패턴의 성별에 따른 차이는 고혈압을 포함한 만성질환의 발생과 진행에 중요한 영향요인으로 작용한다[19]. 본 연구에서는 성별로 구분된 잠재계층에 상이한 생활습관 특징이 포함된 것을 확인하였고, 대사이상 및 비만과 결합된 이질적 하위집단에 따른 임상적 건강특성 차이를 설명하였다.

일반인을 대상으로 생활습관 패턴을 확인한 이전 연구에서 남성은 주로 음주와 흡연이 포함된 패턴을 보였고, 여성은 부족한 신체활동 혹은 이에 다른 요인이 조합된 형태로 분류되는 것을 확인하였다[20]. 본 연구에서 남성 3명 중 2명이 고위험 음주만 포함된 저위험 생활습관 계층에 분포하였고, 여성은 96.5%가 건강위험행동이 없거나 부족한 신체활동만 포함된 저위험 생활습관 계층에 분포하였다. 이처럼 고혈압 환자는 일반인에 비해 저위험 생활습관을 보였다. 특히 여성의 절반이 건강위험행동 요인이 포함되지 않은 계층에 분포하여, 여성 고혈압 환자의 생활습관 개선 경향이 더욱 뚜렷하다고 추측할 수 있다. 이는 이전 연구들에서 높은 혈압을 관리하기 위한 생활습관 개선수준이 여성에서 높다는 결과와 일치하였다[21,22].

또한 항고혈압제를 복용하는 대상자는 생활습관 위험수준과 상관없이 비만인 계층(HB-MO 또는 LB-MO)에 포함될 가능성이 높았다. 선행연구에서도 항고혈압제의 복용이 정상체중을 유지하는 것과 관련이 없었으며, 오히려 약을 복용하는 고혈압 환자가 비만일 확률이 상당히 높다는 결론을 도출하였다[23,24]. 항 고혈압제의 복용과 비만의 관련성은 고혈압 환자의 약에 대한 의존력으로 설명되기도 한다. Kim and Kong(2015)은 항고혈압제의 복용으로 충분히 혈압을 관리할 수 있다는 환자의 생각이 체중 관리를 시도하지 않을 수 있다고 하였다[22]. 반면, 약을 복용하지 않지만 고혈압을 인지한 환자는 생활습관을 개선하여 혈압을 관리하려는 특징이 있다[23]. 유럽 종단연구에서 음주와 흡연상태가 고혈압 인지 여부에 따라 차이를 보였으며, 한국과 스페인에서 실시한 연구 역시 고혈압을 인지하였을 때 비흡연하는 경향이 나타났다[22,25]. 이는 질병의 인지가 건강한 생활습관으로의 이행과 정상체중 유지에 중요한 영향요인으로 작용함을 반영한다. 본 연구에서 고혈압 진단 경험이 없고, 고혈압을 인지하지 못한 대상자가 16.5% 포함되어 있었다. 이들의 효과적인 혈압 관리를 위해서는 고혈압 상태에 대한 인지가 선행되어야 함을 시사하였다.

고혈압의 주요 위험요인으로 알려진 대사 이상과 비만에 대해, 대사적으로 건강하지 않은 정상체중(MUNW)과 대사적으로 건강한 비만(MHO)을 정의하여 고혈압 발생의 단일 효과를 규명한 연구들이 있다[26–29]. 한국인을 대상으로 한 Kang 등(2016)[26]과 Ryoo 등(2017)[27]의 전향적 연구와 중국인을 대상으로 한 Tian 등(2018)[28]의 단면연구에서는 대사적으로 건강한 정상체중(MHNW) 유형에 비해, MHO과 MUNW유형의 고혈압 발생위험이 유의하게 높았으며, 비만과 대사 상태가 고혈압 위험 증가에 독립적인 역할을 하고 있다고 밝혔다. 이에 비해, 본 연구는 고혈압 환자에서 보여지는 군집화된 특징의 확인을 목적으로 하였으며, 모든 계층에 대사이상 상태가 포함되어 대사적으로 건강한 비만 계층이 구분되지 않았다. 코호트 자료를 토대로 고혈압 발생을 추적한 Lee 등(2013)[29]의 연구에서는 8년의 추적기간동안 MHO 집단의 많은 대상자가 대사적으로 건강하지 않은 상태로 전환되었으며, MHO 집단에서 고혈압이 발생한 대상자의 56.5%는 진단 시 대사적으로 건강하지 않은 것을 확인하였다. 이는 비만 여부보다 대사 이상이 고혈압 환자에서 강조되는 본 연구와 유사한 결과로, 고혈압의 발생과 관련된 예측변수로 비만과 대사 이상을 독립적으로 고려할 수 있으나, 고혈압의 관리 측면에서는 대사 이상을 개선하는 것이 더욱 중요하다는 것을 반영한다.

비만과 생활습관 위험행동은 독립적으로 높은 혈압과 관련되어 있다. 여성에서는 생활습관 위험행동에 우선하여 비만이 높은 혈압과 관련되어 있는데 비해, 남성의 경우에는 정상체중인 고위험 생활습관 계층에서도 2단계 고혈압과 유의한 관련을 보였다. 이전 연구들에서는 여성에서 고혈압과 비만의 관련성이 더욱 밀접하다는 사실을 확인하였다[30–32]. Faulkner와 Belin(2018)[32]은 폐경 연령 이후에 여성의 고혈압 위험수준이 높아지며, 폐경 전이더라도 비만한 여성에서 혈압이 BMI와 밀접하게 관련되어, 비만이 높은 혈압과 연관됨을 보고하였다. 한편, 남성에서는 부정적인 건강행동의 영향력이 높았다. 본 연구에서 남성의 모든 점재계층에 흡연력이 포함되었고, 음주 여부에 따라 고위험 생활습관이 정의되어, 고위험과 저위험 생활습관은 음주의 차이로 설명될 수 있었다. Roerecke 등(2018)[33]은 메타분석 결과에서 음주와 고혈압의 관련성이 여성보다 남성에서 유의한 것을 확인하였으며, Choi 등(2017)[34]은 국민건강영양조사 자료에서 남성의 혈압 관리를 방해하는 요인으로 음주를 의미있게 설명하였다. 이와 같이 성별의 차이는 고혈압의 위험요인을 관리하는데 중요한 고려요인이었으며, 여성은 비만을 관리하고, 남성은 비만 뿐 만 아니라 생활습관을 개선하는데 집중할 수 있는 차별화된 접근이 필요하였다. 또한, LB-MO군에는 고혈압의 동반질환이 포함될 가능성이 유의하게 높았으며, 심뇌혈관계 질환 과거력과 고혈압 인지율 역시 높았다. 비록 단면연구의 한계로 조사 시점에서 보인 생활습관 수준이 지속된 것 인지 혹은 일시적인 것인지 명확하지 않지만, 생활습관 수준이 저위험이더라도 비만은 동반질환 발생과 관련성이 있으며, 이로 인하여 치료과정이 복잡해질 수 있다는 것을 확인할 수 있었다.

본 연구에는 몇 가지 제한점이 있다. 첫째, 단면연구 설계의 한계로 고혈압의 인지 및 항고혈압제 복용과 생활습관 변화와의 종단적 관계 뿐 만 아니라, 생활습관 특성과 동반질환 발생 간의 인과적 관련성을 규명할 수 없다. 둘째, 고혈압을 정의하는데 의료기록이 아닌 현재의 혈압상태와 설문조사 응답결과를 기준으로 하였기 때문에, 정확한 진단에 의한 대상자 선정이 이루어지지 못했다. 그러나 다른 측면에서, 진단을 받지 않았으나 고혈압 기준에 속하는 높은 혈압의 대상자를 포함하여, 고혈압을 인지하지 못한 대상자의 상태까지 반영할 수 있었다. 셋째, 본 연구자료는 16개 검진센터에서 수집한 것으로 혈압계 기종이 동일하지 않은 제한점이 있으나, 동일한 지침을 사용하고 주기적인 교육을 통해 혈압측정의 오차를 줄이고자 하였다. 넷째, 일상적으로 건강상태를 확인하는 목적으로 실시한 국가 건강검진 자료를 후향적으로 활용한 점에서 한계가 있다. 16개 검진센터의 지역적 특징이 이질적일 수 있고, 자발적인 검진자를 대상으로 분석한 점에서 자발적 참여에 의한 편향(volunteer bias) 가능성이 있다. 그러나 본 연구자료는 전국에 분포한 검진센터에서 수집되었고, Noh 등(2019)[35]의 연구에서와 같이 국가 건강검진 자료와 비교하여 대표성이 입증되었기 때문에 일반인을 대표한다고 볼 수 있다. 또한 후향적 자료를 수집하여 기존 누적된 데이터 외에 고혈압 관련요인을 분석에 포함하지 못했다. 고혈압의 이환기간은 생활습관 개선정도와 비례하며, 동반질환 위험과도 관련되어 있다[22]. 또한 염분섭취는 고혈압 위험요인으로 알려져 있으며, 금연기간은 혈압과 선형적 관련성이 있다[7,21,36]. 따라서 잠재계층에 이러한 정보를 반영할 수 있다면, 생활습관 개선을 위한 개입 정도와 동반질환 관리를 위한 전략 등 세부적인 접근이 가능할 수 있을 것이다.

본 연구는 대규모의 고혈압 환자 자료를 활용하여 혈압 관리와 밀접하게 관련된 생활습관, 대사 상태 및 비만을 포함하여 군집화된 특징을 규명하였으며, 정의된 잠재계층을 구분짓는 관련요인을 파악한 점에서 의의가 있다. 생활습관 및 비만 여부와 상관없이 모든 잠재계층에 대사이상 상태가 포함된 점은 고혈압과 대사상태의 긴밀한 관련성을 반영하므로, 고혈압 환자를 효과적으로 관리하기 위하여 대사상태를 고려한 접근이 강조되어야 한다. 또한 고혈압 관리를 위한 일반적인 생활 지침과 더불어, 여성은 비만을 관리하고 남성은 비만 뿐 만 아니라 생활습관 개선하는데 노력해야하며, 아울러 생활습관 패턴의 성별에 따른 이질적인 특징을 반영한 차별적인 고혈압 관리가 필요하다.

**Ethics statement**

본 연구는 한국건강관리협회 연구윤리심의위원회의 승인을 받아 진행하였다 (approval IRB no. 130750-201907-HR-020).

**Conflict of interest**

모든 저자는 본 연구에 대해 표명할 이해상충이 없음. (All authors have no conflicts of interest to declare for this study.)

**Funding**

없음. (None.)

**Acknowledgement**

없음. (None.)

**ORCID**

Suyoung Kim: http://orcid.org/0000-0003-0512-1189

Seon Cho: http://orcid.org/0000-0002-6432-5897

Eun-Hee Nah: http://orcid.org/0000-0003-0637-4364

**Reference**

1. World health organization. Hypertension: key facts. [cited 2020 June 16]. Available from: https://www.whCo.int/news-room/fact-sheets/detail/hypertension.

2. Global Burden of Metabolic Risk Factors for Chronic Diseases Collaboration. Cardiovascular disease, chronic kidney disease, and diabetes mortality burden of cardiometabolic risk factors from 1980 to 2010: a comparative risk assessment. Lancet Diabetes Endocrinol 2014; 2: 634–647.

3. Park SM, Lim MK, Shin SA, Yun YH. Impact of prediagnosis smoking, alcohol, obesity, and insulin resistance on survival in male cancer patients: National Health Insurance Corporation Study. J Clin Oncol 2006; 24(31): 5017–5024.

4. Appel LJ, Champagne CM, Harsha DW, Cooper LS, Obarzanek E, Elmer PJ, et al.; Writing Group of the PREMIER Collaborative Research Group. Effects of comprehensive lifestyle modification on blood pressure control: main results of the PREMIER clinical trial. JAMA. 2003;289(16):2083-2093.

5. Nguyen B, Bauman A, Ding D. Association between lifestyle risk factors and incident hypertension among middle-aged and older Australians. Prev Med. 2019;118:73-80.

6. Krokstad S, Ding D, Grunseit AC, Sund ER, Holmen TL, Rangul V, et al. Multiple lifestyle behaviours and mortality, findings from a large population-based Norwegian cohort study - The HUNT Study. BMC Public Health. 2017;17(1):58.

7. Dickinson HO, Mason JM, Nicolson DJ, Campbell F, Beyer FR, Cook JV, et al. Lifestyle interventions to reduce raised blood pressure: a systematic review of randomized controlled trials. J Hypertens. 2006 Feb;24(2):215-233.

8. Alwardat N, Di Renzo L, de Miranda RC, Alwardat S, Sinibaldi Salimei P, De Lorenzo A. Association between hypertension and metabolic disorders among elderly patients in North Jordan. Diabetes Metab Syndr. 2018;12(5):661-666.

9. Ding D, Rogers K, van der Ploeg H, Stamatakis E, Bauman AE. Traditional and Emerging Lifestyle Risk Behaviors and All-Cause Mortality in Middle-Aged and Older Adults: Evidence from a Large Population-Based Australian Cohort. PLoS Med. 2015;12(12):e1001917.

10. Loef M, Walach H. The combined effects of healthy lifestyle behaviors on all cause mortality: a systematic review and meta-analysis. Prev Med. 2012;55(3):163-170.

11. Ingledew DK, Hardy L, Cooper CL. Latent class analysis applied to health behaviours. Pers Individ Dif. 1995;19(1):13–20.

12. Ghanbari J, Mohammadpoorasl A, Jahangiry L, Farhangi MA, Amirzadeh J, Ponnet K. Subgroups of lifestyle patterns among hypertension patients: a latent-class analysis. BMC Med Res Methodol. 2018;18(1):127.

13. Trivedi RB, Ayotte BJ, Thorpe CT, Edelman D, Bosworth HB. Is there a nonadherent subtype of hypertensive patient? A latent class analysis approach. Patient Prefer Adherence. 2010;4:255-262.

14. Mulè G, Calcaterra I, Nardi E, Cerasola G, Cottone S. Metabolic syndrome in hypertensive patients: An unholy alliance. World J Cardiol. 2014;6(9):890-907.

15. The Korean Society of Hypertension. Essentials of 2018 Korean society of hypertension guideline for management of hypertension. [cited 2020 June 16]. Available from: http://www.koreanhypertension. org/reference/guide?mode=read&idno=4246.

16. Hong Y, Chun S, Yun M, Asante LS, Chu C. A study of high-risk drinking patterns among generations based on the 2009 Korea national health and nutrition examination survey. Osong Public Health Res Perspect. 2014;5(1):46-53.

17. World health organization. Physical activity. [cited 2020 June 18]. Available from: https://www.who.int/news-room/fact-sheets/detail/physical-activity.

18. Nylund KL, Asparouhov T, Muthén BO. Deciding on the number of classes in latent class analysis and growth mixture modeling: a Monte Carlo simulation study. Struct Equ Model. 2007;14(4):535–569.

19. Varì R, Scazzocchio B, D'Amore A, Giovannini C, Gessani S, Masella R. Gender-related differences in lifestyle may affect health status. Ann Ist Super Sanita. 2016;52(2):158-166.

20. John U, Hanke M, Freyer-Adam J. Health Risk Behavior Patterns in a National Adult Population Survey. Int J Environ Res Public Health. 2018;15(5):873.

21. Hu H, Li G, Arao T. Prevalence rates of self-care behaviors and related factors in a rural hypertension population: a questionnaire survey. Int J Hypertens. 2013;2013:526949.

22. Kim Y, Kong KA. Do hypertensive individuals who are aware of their disease follow lifestyle recommendations better than those who are not aware? PLoS One. 2015;10(8):e0136858.

23. Akbarpour S, Khalili D, Zeraati H, Mansournia MA, Ramezankhani A, Fotouhi A. Healthy lifestyle behaviors and control of hypertension among adult hypertensive patients. Sci Rep. 2018;8(1):8508.

24. Neutel CI, Campbell N, Canadian Hypertension S. Changes in lifestyle after hypertension diagnosis in Canada. Can J Cardiol. 2008; 24(3):199–204.

25. Gee ME, Bienek A, Campbell NR, Bancej CM, Robitaille C, Kaczorowski J, et al. Prevalence of, and barriers to, preventive lifestyle behaviors in hypertension (from a national survey of Canadians with hypertension). Am J Cardiol. 2012;109(4):570-575.

26. Kang YM, Jung CH, Jang JE, Hwang JY, Kim EH, Park JY, et al. The association of incident hypertension with metabolic health and obesity status: definition of metabolic health does not matter. Clin Endocrinol (Oxf). 2016;85(2):207-215.

27. Ryoo JH, Park SK, Oh CM, Choi YJ, Chung JY, Ham WT, et al. Evaluating the risk of hypertension according to the metabolic health status stratified by degree of obesity. J Am Soc Hypertens. 2017;11(1):20-27.e4.

28. Tian S, Xu Y, Dong H. The effect of metabolic health and obesity phenotypes on risk of hypertension: A nationwide population-based study using 5 representative definitions of metabolic health. Medicine (Baltimore). 2018;97(38):e12425.

29. Lee SK, Kim SH, Cho GY, Baik I, Lim HE, Park CG, et al. Obesity phenotype and incident hypertension: a prospective community-based cohort study. J Hypertens. 2013;31(1):145-151.

30. Fujita M, Hata A. Sex and age differences in the effect of obesity on incidence of hypertension in the Japanese population: a large historical cohort study. J Am Soc Hypertens. 2014;8:64–70.

31. Sampson UK, Edwards TL, Jahangir E, Munro H, Wariboko M, Wassef MG, et al. Factors associated with the prevalence of hypertension in the southeastern United States: insights from 69,211 blacks and whites in the Southern Community Cohort Study. Circ Cardiovasc Qual Outcomes. 2014;7:33–54.

32. Faulkner JL, Belin de Chantemèle EJ. Sex Differences in Mechanisms of Hypertension Associated With Obesity. Hypertension. 2018;71(1):15-21.

33. Roerecke M, Tobe SW, Kaczorowski J, Bacon SL, Vafaei A, Hasan OSM, et al. Sex-Specific Associations Between Alcohol Consumption and Incidence of Hypertension: A Systematic Review and Meta-Analysis of Cohort Studies. J Am Heart Assoc. 2018;7(13):e008202.

34. Choi HM, Kim HC, Kang DR. Sex differences in hypertension prevalence and control: Analysis of the 2010-2014 Korea National Health and Nutrition Examination Survey. PLoS One. 2017;12(5):e0178334.

35. Noh Y, Jeong HE, Kim HJ, Ko H, Nah EH and Shin JY. The Multi-Institutional Health Screening Records Database of South Korea: Description and Evaluation of Its Characteristics. Yonsei Med J. 2019;60(12):1216-1222.

36. Halimi JM, Giraudeau B, Vol S, Cacès E, Nivet H, Tichet J. The risk of hypertension in men: direct and indirect effects of chronic smoking. J Hypertens. 2002;20(2):187-193.

**Figure Legend**

Figure 1. Flow diagram for study participants.

Table 1. Characteristics of study subjects according to sex

|  | | **Men (N=102,780)** | |  | **Women (N=103,710)** | |  | **p-value** |
| --- | --- | --- | --- | --- | --- | --- | --- | --- |
|  |  | Mean ± | SD |  | Mean ± | SD |  |  |
| **Age (yrs)** | | 61.9 ± | 10.5 |  | 64.6 ± | 9.2 |  | <0.001 |
| **Anthropometric measurement** | |  |  |  |  |  |  |  |
|  | SBP, mmHg | 132.7 ± | 15.1 |  | 132.9 ± | 15.7 |  | 0.077 |
|  | DBP, mmHg | 82.3 ± | 10.7 |  | 79.9 ± | 10 |  | <0.001 |
|  | Waist, cm | 88.7 ± | 13.9 |  | 83.8 ± | 11.2 |  | <0.001 |
|  | BMI, kg/m² | 25.6 ± | 3.1 |  | 25.5 ± | 3.5 |  | <0.001 |
| **Clinical factors** | |  |  |  |  |  |  |  |
|  | TG, mg/dL | 155.8 ± | 117.9 |  | 127 ± | 77.5 |  | <0.001 |
|  | HDL cholesterol, mg/dL | 49.6 ± | 12 |  | 55.3 ± | 12.8 |  | <0.001 |
|  | LDL cholesterol, mg/dL | 105.6 ± | 35.8 |  | 111.8 ± | 36.6 |  | <0.001 |
|  | FBS, mg/dL | 112.9 ± | 30 |  | 107.1 ± | 26.1 |  | <0.001 |
|  | AST, IU/L | 30.9 ± | 21.7 |  | 27.7 ± | 16.6 |  | <0.001 |
|  | ALT, IU/L | 30.4 ± | 25.4 |  | 24 ± | 19.3 |  | <0.001 |
|  | γ-GTP, IU/L | 60.4 ± | 88.9 |  | 30.8 ± | 40.3 |  | <0.001 |
|  | Serum creatinine, mg/dL | 1.12 ± | 0.38 |  | 0.86 ± | 0.27 |  | <0.001 |
|  | e-GFR, mL/min/1.73m² | 74.9 ± | 15.8 |  | 74.5 ± | 15.8 |  | <0.001 |
|  | Urine protein(+), n(%) | 7,399 | (7.2) |  | 3,938 | (3.8) |  | <0.001 |
| **History of hypertension, n(%)** | |  |  |  |  |  |  |  |
|  | No awareness | 18,947 | (18.4) |  | 15,075 | (14.5) |  | <0.001 |
|  | Diagnosis | 4,257 | (4.1) |  | 2,581 | (2.5) |  |  |
|  | Diagnosis+Medication | 79,574 | (77.4) |  | 86,054 | (83.0) |  |  |
| **History of disease, n(%)** | |  |  |  |  |  |  |  |
|  | Overall | 10,567 | (10.3) |  | 8,026 | (7.7) |  | <0.001 |
|  | Stroke | 3,746 | (3.6) |  | 2,870 | (2.8) |  | <0.001 |
|  | Heart disease | 7,426 | (7.2) |  | 5,570 | (5.4) |  | <0.001 |
| **Family history of hypertension, n(%)** | | 29,958 | (29.2) |  | 35,483 | (34.2) |  | <0.001 |
| **Latent class indicators, n(%)** | |  |  |  |  |  |  |  |
| **Lifestyle behavior** | |  |  |  |  |  |  |  |
|  | Uncontrolled blood pressure | 45,286 | (44.1) |  | 41,918 | (40.4) |  | <0.001 |
|  | Smoking: Past or current | 74,786 | (72.8) |  | 3,797 | (3.7) |  | <0.001 |
|  | High-risk alcohol drinking | 33,129 | (32.2) |  | 3,546 | (3.4) |  | <0.001 |
|  | Insufficient physical activity | 43,495 | (42.3) |  | 53,636 | (51.7) |  | <0.001 |
| **Metabolic status** | |  |  |  |  |  |  |  |
|  | Metabolic risk (≥1 component) | 85,222 | (82.9) |  | 84,121 | (81.1) |  | <0.001 |
| **Obesity** | |  |  |  |  |  |  |  |
|  | Obesity | 57,594 | (56.0) |  | 53,875 | (52.0) |  | <0.001 |
|  | Abdominal obesity | 43,231 | (42.1) |  | 44,831 | (43.2) |  | <0.001 |

Abbreviations: SBP, systolic blood pressure; DBP, diastolic blood pressure; TG, triglycerides; HDL, high density lipoprotein; LDL, low density lipoprotein; FBS, fasting blood glucose; AST, aspartate transaminase; ALT, alanine transaminase; γ-GTP, gamma-glutamic transpeptidase; e-GFR, estimated glomerular filtration rate

Table 2. Comparison of model fit statistics for the latent class models by sex

| **Sex** | **Classes** | **Log-Likelihood** | **G²** | **AIC** | **BIC** | **CAIC** | **adj. BIC** | **Entropy** | **df** |
| --- | --- | --- | --- | --- | --- | --- | --- | --- | --- |
| **Male** |  |  |  |  |  |  |  |  |  |
|  | 2 | -431603.15 | 4081.38 | 4111.38 | 4254.48 | 4269.48 | 4206.81 | 0.72 | 112 |
|  | 3 | -430447.66 | 1770.39 | 1816.39 | 2035.82 | 2058.82 | 1962.73 | 0.82 | 104 |
|  | **4** | **-429942.79** | **760.65** | **822.65** | **1118.4** | **1149.4** | **1019.88** | **0.83** | **96** |
|  | 5 | -426848.17 | 534.55 | 612.55 | 984.62 | 1023.62 | 860.68 | 0.71 | 88 |
|  | 6 | -429671.73 | 218.53 | 312.53 | 760.93 | 807.93 | 611.56 | 0.65 | 80 |
| **Female** |  |  |  |  |  |  |  |  |  |
|  | 2 | -344564.29 | 2844.28 | 2874.28 | 3017.52 | 3032.52 | 2969.85 | 0.75 | 112 |
|  | 3 | -343756.32 | 1228.34 | 1274.34 | 1493.98 | 1516.98 | 1420.89 | 0.84 | 104 |
|  | **4** | **-343462.67** | **641.03** | **703.03** | **999.06** | **1030.06** | **900.54** | **0.87** | **96** |
|  | 5 | -343285.91 | 287.51 | 365.51 | 737.94 | 776.94 | 613.99 | 0.74 | 88 |
|  | 6 | -343227.77 | 171.24 | 265.24 | 714.06 | 761.06 | 564.69 | 0.72 | 80 |

Abbreviations: AIC, Akaike information criterion; BIC, Bayesian information criterion; CAIC, Consistent AIC; adj. BIC, Adjusted BIC; df, degree of freedom.

Table 3. Prevalence of lifestyle behaviors, metabolic risk, and obesity indicators, and conditional probabilities of class membership in the class mode

|  | | **Latent class** | | | |
| --- | --- | --- | --- | --- | --- |
|  |  | **Class Ⅰ (HB-MO)** | **Class Ⅱ (LB-MO)** | **Class Ⅲ (HB-MNW)** | **Class Ⅳ (LB-MNW)** |
| **Male** | |  |  |  |  |
|  | Latent class prevalence | 16.9% | 32.4% | 15.3% | 35.5% |
|  |  |  |  |  |  |
| **Lifestyle behaviors** | |  |  |  |  |
|  | Uncontrolled blood pressure | 0.484 | 0.437 | 0.469 | 0.411 |
|  | Past or current smoking | **0.829** | **0.695** | **0.842** | **0.660** |
|  | High-risk alcohol drinking | **0.983** | 0.011 | **0.994** | 0.002 |
|  | Insufficient physical activity | 0.441 | 0.454 | 0.405 | 0.394 |
| **Metabolic status** | |  |  |  |  |
|  | Metabolic risk (≥1 component) | **0.902** | **0.878** | **0.801** | **0.762** |
| **Obesity status** | |  |  |  |  |
|  | Obesity (BMI≥25 kg/m²) | **0.937** | **0.918** | 0.229 | 0.197 |
|  | Abdominal obesity  (WC≥90 cm, men; WC≥85 cm, women) | **0.843** | **0.821** | 0.026 | 0.024 |
|  |  |  |  |  |  |
| **Female** | |  |  |  |  |
|  | Latent class prevalence | 1.7% | 47.1% | 1.8% | 49.4% |
|  |  |  |  |  |  |
| **Lifestyle behaviors** | |  |  |  |  |
|  | Uncontrolled blood pressure | **0.510** | 0.418 | 0.326 | 0.390 |
|  | Past or current smoking | 0.209 | 0.034 | **0.961** | 0.000 |
|  | High-risk alcohol drinking | **0.974** | 0.000 | 0.255 | 0.026 |
|  | Insufficient physical activity | **0.558** | **0.560** | **0.550** | 0.474 |
| **Metabolic status** | |  |  |  |  |
|  | Metabolic risk (≥1 component) | **0.797** | **0.878** | **0.751** | **0.750** |
| **Obesity status** | |  |  |  |  |
|  | Obesity (BMI≥25 kg/m²) | **0.891** | **0.888** | 0.067 | 0.171 |
|  | Abdominal obesity  (WC≥90 cm, men; WC≥85 cm, women) | **0.872** | **0.856** | 0.041 | 0.027 |

Abbreviations: HP-MO, High risk lifestyle behavior with metabolic disorder and obesity; LP-MO, Low risk lifestyle behavior with metabolic disorder and obesity; HP-MNW, High risk lifestyle behavior with metabolic disorder and normal weight; LP-MNW, Low risk lifestyle behavior with metabolic disorder and normal weight; BMI, body mass index; WC, waist circumference.

Table 4. Differences in characteristics of anthropometric measurements, clinical factors, and disease history among the four latent classes

|  | | | **Class Ⅰ (HB-MO)** | | **Class Ⅱ (LB-MO)** | | **Class Ⅲ (HB-MNW)** | | **Class Ⅳ (LB-MNW)** | | **p-value** | **Multiple  comparison** |
| --- | --- | --- | --- | --- | --- | --- | --- | --- | --- | --- | --- | --- |
| **Male** | | |  |  |  |  |  |  |  |  |  |  |
|  | **Age(yrs)** | | 57.3 ± | 9.6 | 62.5 ± | 10.8 | 58.6 ± | 9.1 | 64.7 ± | 10.1 | <0.001 | Ⅰ<Ⅲ<Ⅱ<Ⅳ |
|  | **Anthropometric measurement** | | |  |  |  |  |  |  |  |  |  |
|  |  | SBP, mmHg | 133.5 ± | 15 | 132.9 ± | 14.9 | 132.9 ± | 15.4 | 132.3 ± | 15.3 | <0.001 | Ⅳ<Ⅱ,Ⅲ<Ⅰ |
|  |  | DBP, mmHg | 84.5 ± | 10.7 | 82.2 ± | 10.5 | 83.7 ± | 10.8 | 81 ± | 10.5 | <0.001 | Ⅳ<Ⅱ<Ⅲ<Ⅰ |
|  | **History of HTN, n(%)** | |  |  |  |  |  |  |  |  |  |  |
|  |  | No awareness | 2,867 | (19.0) | 4,625 | (15) | 4,457 | (24.8) | 6,998 | (18.0) | <0.001 |  |
|  |  | Diagnosis | 695 | (4.6) | 983 | (3.2) | 953 | (5.3) | 1,626 | (4.2) |  |  |
|  |  | Diagnosis+Medication | 11,562 | (76.5) | 25,150 | (81.8) | 12,595 | (70.0) | 30,267 | (77.8) |  |  |
|  | **History of disease, n(%)** | |  |  |  |  |  |  |  |  |  |  |
|  |  | Overall | 934 | (6.2) | 3906 | (12.7) | 1003 | (5.6) | 4724 | (12.2) | <0.001 |  |
|  |  | Stroke | 282 | (1.9) | 1347 | (4.4) | 322 | (1.8) | 1795 | (4.6) | <0.001 |  |
|  |  | Heart disease | 698 | (4.6) | 2798 | (9.1) | 705 | (3.9) | 3225 | (8.3) | <0.001 |  |
|  | **Family history of HTN, n(%)** | | 6,445 | (42.6) | 11,665 | (37.9) | 7,039 | (39.1) | 13,769 | (35.4) | <0.001 |  |
|  | **Comorbidities** | |  |  |  |  |  |  |  |  |  |  |
|  |  | Stage 2 HTN, n(%) | 1,440 | (9.5) | 2,311 | (7.5) | 1,704 | (9.5) | 2,681 | (6.9) | <0.001 |  |
|  |  | Diabetes, n(%) | 5,458 | (36.1) | 11,758 | (38.2) | 5,370 | (29.8) | 12,133 | (31.2) | <0.001 |  |
|  |  | Renal dysfunction, n(%) | 2,333 | (15.4) | 6,955 | (22.6) | 2,253 | (12.5) | 7,525 | (19.4) | <0.001 |  |
|  |  | Liver dysfunction, n(%) | 7,273 | (48.1) | 8,161 | (26.5) | 6,414 | (35.6) | 5,411 | (13.9) | <0.001 |  |
| **Female** | | |  |  |  |  |  |  |  |  |  |  |
|  | **Age (yrs)** | | 56 ± | 8.7 | 65.5 ± | 9.2 | 59.5 ± | 9.8 | 64.2 ± | 9.1 | <0.001 | Ⅰ<Ⅲ<Ⅳ<Ⅱ |
|  | **Anthropometric measurement** | | |  |  |  |  |  |  |  |  |  |
|  |  | SBP, mmHg | 135.2 ± | 16.1 | 134.6 ± | 15.5 | 127.7 ± | 16.4 | 131.5 ± | 15.6 | <0.001 | Ⅲ<Ⅳ<Ⅱ<Ⅰ |
|  |  | DBP, mmHg | 84.7 ± | 10.8 | 80.5 ± | 9.8 | 79.1 ± | 11 | 79.4 ± | 10 | <0.001 | Ⅲ,Ⅳ<Ⅱ<Ⅰ |
|  | **History of HTN, n(%)** | |  |  |  |  |  |  |  |  |  |  |
|  |  | No awareness | 407 | (23.6) | 5891 | (12.9) | 263 | (16.5) | 8,514 | (15.6) | <0.001 |  |
|  |  | Diagnosis | 49 | (2.8) | 864 | (1.9) | 76 | (4.8) | 1,592 | (2.9) |  |  |
|  |  | Diagnosis+Medication | 1,272 | (73.6) | 39,044 | (85.3) | 1,258 | (78.8) | 44,480 | (81.5) |  |  |
|  | **History of disease, n(%)** | |  |  |  |  |  |  |  |  |  |  |
|  |  | Overall | 64 | (3.7) | 4,027 | (8.8) | 115 | (7.2) | 3,820 | (7.0) | <0.001 |  |
|  |  | Stroke | 22 | (1.3) | 1,403 | (3.1) | 50 | (3.1) | 1,395 | (2.6) | <0.001 |  |
|  |  | Heart disease | 46 | (2.7) | 2,829 | (6.2) | 73 | (4.6) | 2,622 | (4.8) | <0.001 |  |
|  | **Family history of HTN, n(%)** | | 856 | (49.5) | 18,509 | (40.4) | 799 | (50.0) | 24001 | (44.0) | <0.001 |  |
|  | **Comorbidities** | |  |  |  |  |  |  |  |  |  |  |
|  |  | Stage 2 HTN, n(%) | 199 | (11.5) | 3,164 | (6.9) | 89 | (5.6) | 3,065 | (5.6) | <0.001 |  |
|  |  | Diabetes, n(%) | 401 | (23.2) | 15,427 | (33.7) | 349 | (21.9) | 12,364 | (22.7) | <0.001 |  |
|  |  | Renal dysfunction, n(%) | 148 | (8.6) | 9,773 | (21.3) | 227 | (14.2) | 9,296 | (17.0) | <0.001 |  |
|  |  | Liver dysfunction, n(%) | 714 | (41.3) | 9,202 | (20.1) | 406 | (25.4) | 6,309 | (11.6) | <0.001 |  |

1. Data were described as mean ± SD or n(%).

Abbreviations: HP-MO, High risk lifestyle behavior with metabolic disorder and obesity; LP-MO, Low risk lifestyle behavior with metabolic disorder and obesity; HP-MNW, High risk lifestyle behavior with metabolic disorder and normal weight; LP-MNW, Low risk lifestyle behavior with metabolic disorder and normal weight; SBP, systolic blood pressure; DBP, diastolic blood pressure; e-GFR, estimated glomerular filtration rate; HTN, hypertension.

Table 5. Associations between latent class membership and demographic or clinical characteristics

|  | | | **Latent class** | | | | | | | |
| --- | --- | --- | --- | --- | --- | --- | --- | --- | --- | --- |
|  |  |  | **Class Ⅰ (HB-MO)** | | **Class Ⅱ (LB-MO)** | | **Class Ⅲ (HB-MNW)** | | **Class Ⅳ (LB-MNW)** |  |
|  |  |  | OR | (95% CI) | OR | (95% CI) | OR | (95% CI) | Reference |  |
| **Male** | | |  |  |  |  |  |  |  |  |
|  | Age (per 10-year increase) | | 0.53 | (0.52, 0.54)* | 0.79 | (0.78, 0.81)* | 0.62 | (0.61, 0.63)* | 1 |  |
|  | History of disease | | 0.64 | (0.59, 0.69)* | 1.08 | (1.03, 1.13)* | 0.58 | (0.54, 0.62)* | 1 |  |
|  | Medication for hypertension | | 1.50 | (1.43, 1.58)* | 1.43 | (1.37, 1.49)* | 0.99 | (0.95, 1.04) | 1 |  |
|  | Family history of hypertension | | 1.02 | (0.98, 1.06) | 0.99 | (0.96, 1.02) | 0.97 | (0.93, 1.00) | 1 |  |
|  | Comorbidities | |  |  |  |  |  |  |  |  |
|  |  | Stage 2 Hypertension | 1.21 | (1.12, 1.30)* | 1.14 | (1.08, 1.22)* | 1.09 | (1.02, 1.17)* | 1 |  |
|  |  | Diabetes | 1.41 | (1.35, 1.47)* | 1.33 | (1.29, 1.37)* | 1.12 | (1.07, 1.16)* | 1 |  |
|  |  | Renal dysfunction | 0.95 | (0.90, 1.00) | 1.26 | (1.21, 1.30)* | 0.76 | (0.72, 0.80)* | 1 |  |
|  |  | Liver dysfunction | 4.32 | (4.13, 4.51)* | 2.03 | (1.96, 2.11)* | 2.72 | (2.61, 2.84)* | 1 |  |
| **Female** | | |  |  |  |  |  |  |  |  |
|  | Age (per 10-year increase) | | 0.40 | (0.38, 0.43)* | 1.12 | (1.11, 1.14)* | 0.58 | (0.55, 0.61)* | 1 |  |
|  | History of disease | | 0.85 | (0.66, 1.10) | 1.12 | (1.07, 1.17)* | 1.36 | (1.12, 1.65)* | 1 |  |
|  | Medication for hypertension | | 1.19 | (1.05, 1.35)* | 1.18 | (1.14, 1.23)* | 1.10 | (0.96, 1.25) | 1 |  |
|  | Family history of hypertension | | 0.92 | (0.83, 1.01) | 0.91 | (0.88, 0.93)* | 1.04 | (0.94, 1.16) | 1 |  |
|  | Comorbidities | |  |  |  |  |  |  |  |  |
|  |  | Stage 2 Hypertension | 1.74 | (1.47, 2.05)* | 1.34 | (1.27, 1.42)* | 0.89 | (0.71, 1.11) | 1 |  |
|  |  | Diabetes | 1.15 | (1.02, 1.29)* | 1.57 | (1.52, 1.61)* | 1.00 | (0.89, 1.13) | 1 |  |
|  |  | Renal dysfunction | 0.70 | (0.58, 0.83)* | 1.14 | (1.10, 1.18)* | 1.06 | (0.92, 1.23) | 1 |  |
|  |  | Liver dysfunction | 4.42 | (4.00, 4.90)* | 1.90 | (1.83, 1.97)* | 2.32 | (2.06, 2.61)* | 1 |  |

Abbreviations: HP-MO, High risk lifestyle behavior with metabolic disorder and obesity; LP-MO, Low risk lifestyle behavior with metabolic disorder and obesity; HP-MNW, High risk lifestyle behavior with metabolic disorder and normal weight; LP-MNW, Low risk lifestyle behavior with metabolic disorder and normal weight; OR, odds ratio; CI, confidence interval.

* p < 0.05.
